# Cancer Premature Mortality Costs in Europe in 2020: A Comparison of the Human Capital Approach and the Friction Cost Approach

**DOI:** 10.3390/curroncol29050287

**Published:** 2022-05-13

**Authors:** Paul Hanly, Marta Ortega-Ortega, Isabelle Soerjomataram

**Affiliations:** 1 School of Business, National College of Ireland, Dublin 1, Ireland; 2 Department of Applied Economics, Public Economics and Political Economy, Faculty of Economics and Business, Complutense University of Madrid, Campus de Somosaguas, 28223 Madrid, Spain; martao@ucm.es; 3 International Agency for Research on Cancer, 69372 Lyon, France; soerjomataraml@iarc.fr

**Keywords:** societal perspective, cancer, productivity costs, human capital approach, friction cost approach, economic evaluation

## Abstract

The inclusion of productivity costs can affect the outcome of cost-effectiveness analyses. We estimated the value of cancer premature mortality productivity costs for Europe in 2020 using the Human Capital Approach (HCA) and compared these to the Friction Cost Approach (FCA). Cancer mortality data were obtained from GLOBOCAN 2020 by sex and five-year age groups. Twenty-three cancer sites for 31 European countries were included. The HCA and the FCA were valued using average annual gross wages by sex and age group and applied to Years of Potential Productive Life Lost. 2020 friction periods were calculated and all costs were in 2020 euros. Estimated cancer premature mortality costs for Europe in 2020 were EUR 54.0 billion (HCA) and EUR 1.57 billion (FCA). The HCA/FCA cost ratio for Europe was 34.4, but considerable variation arose across countries (highest in Ireland: 64.5 v lowest in Czech Republic: 11.1). Both the HCA and the FCA ranked lung, breast and colorectal as the top three most costly cancers in Europe, but cost per death altered rankings substantially. Significant cost differences were observed following sensitivity analysis. Our study provides a unique perspective of the difference between HCA and FCA estimates of productivity costs by cancer site and country in Europe.

## 1. Introduction

Cancer is a leading cause of death globally, accounting for almost 10 million deaths in 2020, of which 20% arose in Europe [1]. This public health burden translates into a substantial economic and societal burden, which amounted to almost EUR 200 billion in Europe in 2018, consisting of both direct costs (cancer-specific health expenditure including expenditure on drugs) and indirect costs (premature mortality, morbidity and informal care costs) [2]. A considerable portion of the total cost burden of cancer is accounted for by productivity losses, of which premature mortality costs comprise the largest share.

In the vast majority of published cost of illness studies, productivity costs have been evaluated according to the Human Capital Approach (HCA) [3]; however, the Friction Cost Approach (FCA) offers an alternative [3,4]. The FCA tends to be more prevalent in Canada, Netherlands and Germany, which were early adopters and endorsers of including FCA estimated costs in their national pharmacoeconomic guidelines. The FCA is predicated on the assumption that unemployment levels above a frictional level exist in many economies, and so employees lost from work through illness and premature mortality can be replaced from a pool of the unemployed [5]. The productivity loss to the economy, and society at large, is therefore assumed to be transitory and smaller than the HCA equivalent cost, which measures productivity costs over the course of an entire working life cut short by premature death.

Most cancer-related premature mortality productivity cost studies to date apply the HCA to value productivity loss [6]. Indeed, only 2 European studies from a review of 17 studies included cancer mortality FCA-derived productivity costs, and even then, they were presented in addition to comparator HCA estimates [6]. More general reviews of the applied cost of illness literature have shown that productivity costs estimated according to the FCA are smaller than the equivalent HCA estimates across disease type, but that the relative magnitudes vary widely, and the divergence is greater for chronic illnesses [3]. Of the reviewed studies that focused on cancer mortality, HCA/FCA cost ratios ranged from 24 to 48 [3].

Although a number of studies in the literature have attempted to explore the differences between cancer-related productivity costs estimated according to the HCA and the FCA, these were limited to single, or just a few, cancer sites and located in a single country [7,8,9,10]. No previous study has attempted to estimate FCA derived productivity costs associated with cancer premature mortality in Europe across multiple sites using a standardized methodological approach to friction period estimation and directly compared these to the alternative HCA. Our study is therefore unique and aims to estimate the value of productivity losses due to cancer-related premature mortality across Europe in 2020 according to the HCA and FCA, and compare estimates by country, sex and cancer site.

## 2. Materials and Methods

### 2.1. Mortality Data and Approach

Cancer mortality data consisted of the number of deaths for each cancer site by sex and five-year age groups (from 15–64 years where deaths were assumed to occur at the midpoint of each 5-year age group) and was obtained from the Global Cancer Observatory, GLOBOCAN 2020 database (https://gco.iarc.fr/today/data-sources-methods, accessed on 4 March 2022). Twenty-three individual cancer sites in addition to a total for all cancer sites were considered following the International Classification of Diseases, 10th revision (ICD-10). We included 31 European countries (the 27 countries of the European Union (EU), plus Norway, Switzerland, Iceland and the United Kingdom (UK)), which we hereafter refer to as ‘Europe,’ and four European regions (Northern, Southern, Central-Eastern, Western Europe).

Our estimates focused on paid production productivity loss, defined by Ortega-Ortega et al. [11] as the monetary value produced by a person who is working in the labour market. Therefore, we estimated the productivity lost in the productive age group, assumed to be between 15–64 years old considering the average statutory retirement in Europe at age 65 and in line with previous premature mortality cost studies in Europe [11,12].

Years of potential productive life lost (YPPLL) were calculated using the Standard Lifetable from the World Health Organisation (WHO) (https://www.who.int/data/gho/data/themes/topics/indicator-groups/indicator-group-details/GHO/gho-ghe-global-health-estimates-life-tables, accessed on 3 March 2022), which reflects the years of potential production that have been lost due to each premature cancer death before 65 years of age by sex and country (YPPLL = life expectancy up to 65 − age at cancer death). YPPLL were subsequently multiplied by market annual gross wages by age group, sex and country to generate productivity costs according to two valuation methods.

#### 2.1.1. Human Capital Approach (HCA)

The HCA is the method most commonly used in the literature to estimate labour productivity lost. It estimates the monetary value of a stream of output over a working lifetime that is cut short by premature death [13] and therefore is an estimate of the potential labour productivity lost when a person dies prematurely.

We valued productivity lost using average annual gross wages by sex, age group (15–29, 30–49, 50–59, 60–64 years) and country sourced from the Structure of Earnings Survey 2018 [13] and inflated to 2020 values using the Harmonized Index of Consumer Price [14], and accounted for country-specific macroeconomic conditions using national unemployment rates for 2020 [15] and labour force participation rates for 2020 [16], disaggregated by sex and age group (15–29, 30–39, 40–49, 50–59, 60–64 years). Present and future flows of potential productivity lost were valued by multiplying YPPLL by the sex-, age- and macroeconomic condition-adjusted wages for each country.

We assumed that wages grew at the average Gross Domestic Product (GDP) growth rate between 2000 to 2020 for each European country (base case) [17], and future costs were discounted at 3.5% per annum (base-case). All costs were expressed in 2020 euros.

#### 2.1.2. Friction Cost Approach

The estimation of the FCA required an estimate of the friction period (the period of time to restore the production level of a firm following the loss of an employee and to fill a vacancy that has arisen) and monetary valuation data for lost production to the economy. Our estimate of country-specific friction periods, updated to 2020, followed a recently published methodology [18]. Initially, the average vacancy duration following the loss of an employee was calculated according to:

$$\mathrm{Annual}\;\mathrm{vacancy}\;\mathrm{duration}\;(\mathrm{VD})=365\;*\;\left[\frac{\sum_{i=1}^{4}\frac{Vi}{4}}{\sum_{i=1}^{4}Mi}\right]$$

where

*V* = stock of unfilled vacancies
*M* = flows of filled vacancies

Estimates for the stock of unfilled vacancies were derived from annual Labour Force Surveys reported by Eurostat in 2020 [19]. Vacancy stock data are missing for France, Italy, Denmark, Hungary and Malta. In the case of Italy, Denmark, Hungary and Malta, we used average friction periods from each of their European regions and used these as approximate estimates of the friction period. For France, and following Hanly et al. [18], we used proxy estimates for vacancy stock (https://tradingeconomics.com/france/job-vacancies, accessed on 10 January 2022). Estimates of the annual flows of filled vacancies per country were calculated by multiplying the total number of occupied posts in each country [20] by the percentage of newly occupied posts in the last 12 months in that country [21]. Following previous studies, we added an additional 4 weeks to the country-specific vacancy duration periods [18], and we assumed an elasticity of annual labour time versus annual labour productivity of 1 [18].

Using the same YPPLL data as for the HCA, we valued cancer-related premature mortality by multiplying YPPLL by country-specific friction periods and by country-, sex- and age-specific average annual gross 2020 wages, following adjustments for country-, sex- and age-specific unemployment and labour force participation rates.

### 2.2. Sensitivity Analysis

Separate sensitivity analyses were conducted for the HCA and FCA. For the HCA, a sensitivity analysis was conducted using maximum and minimum country-specific GDP growth between 2000 and 2020 and 0% and 6% discount rates. For the FCA, a sensitivity analysis was run on maximum and minimum estimates of the friction period between 2008 and 2018 based on results reported by Hanly et al. [18] and on an estimate of the friction period for 2018, prior to the outbreak of COVID-19, for comparative purposes. Excel and Stata (version 18, StataCorp LLC, College Station, TX, USA) were used for analysis.

## 3. Results

### 3.1. Cancer Mortality and Years of Potential Productive Life Lost (YPPLL) in Europe

As shown in Table 1, a total of 336,964 premature cancer-related deaths (15–64 years of age) were estimated for 2020 across Europe, which amounted to 2,833,925 YPPLL. Of these deaths, Western Europe accounted for the largest portion (36%), followed by Southern Europe (25%), Central-Eastern Europe (24%) and Northern Europe (16%).

### 3.2. Total Premature Mortality Costs by Region and Country

In Table 1, the total estimated cancer premature mortality cost for Europe in 2020 was EUR 54.02 billion according to the HCA and EUR 1.57 billion according to the FCA, with HCA costs therefore 34.4 times the size of the equivalent FCA costs. Across the four European regions, this ratio varies from 29.7 in Western Europe (HCA = EUR 27.18 billion; FCA = EUR 0.92 billion) to 49.9 in Southern Europe (HCA = EUR 10.62 billion; FCA = EUR 0.21 billion).

From a country-specific perspective, the ranking of premature mortality costs remained similar between the HCA and FCA, with four (Germany, United Kingdom, France and Italy) of the top five ranked countries by cost burden being the same regardless of valuation approach employed (Spain ranks as fifth most burdensome by cancer cost in Europe according to the HCA, but eighth according to the FCA). Despite this degree of relative similarity in the ranking of the cancer cost burden by country, there was considerable variation in the HCA/FCA cost ratio across countries. The highest cost ratios were recorded for Ireland (64.5), Spain (63.5) and Portugal (60.0), while the lowest arose in Germany (24.3), Belgium (20.2) and Czechia (11.1).

### 3.3. Premature Mortality Costs per Cancer Death by Region and Country

The estimated cancer premature mortality cost per death for Europe in 2020 was EUR 160,318 according to the HCA and EUR 4656 according to the FCA (Table 1). Although the HCA/FCA cost ratios were the same as those for total premature mortality cost, the ranking of the cancer cost burden by country changed considerably. The top three costliest countries for cancer according to the HCA were Switzerland (EUR 456,884), Denmark (EUR 300,750) and Iceland (EUR 281,944). Only Switzerland (EUR 9276) remained in the top three costliest countries according to the FCA, with Belgium (EUR 10,387: 1st) and Germany (EUR 9221: 3rd) emerging as high-cost countries using this approach. Likewise, differences emerged in the ranking of the least costly countries with Croatia (EUR 50,441), Romania (EUR 49,208) and Bulgaria (EUR 46,526) ranked lowest according to the HCA, while Poland (EUR 1236), Lithuania (EUR 1138) and Bulgaria (EUR 988) were lowest ranked by the FCA.

### 3.4. Premature Mortality Costs by Gender, by Region and Country

The total estimated premature mortality cancer cost for Europe in 2020 was EUR 33.81 billion (EUR 176,787 per death) for males and EUR 20.20 billion (EUR 138,686 per death) for females according to the HCA (Table 2). The equivalent cost for the FCA was EUR 1.02 billion (EUR 5331 per death) for males and EUR 0.55 billion (EUR 3771 per death) for females, which resulted in a HCA/FCA cost ratio of 33.2 for male cancer costs and 36.8 for female cancer costs. By region, the highest HCA/FCA cost ratio arose in Southern Europe, where HCA costs were 48.3 times higher for males and 53.0 times higher for females, while the lowest ratio occurred in Western Europe where HCA costs were 28.7 times higher for males and 31.7 times higher for females. This higher HCA/FCA cost ratio for females occurred across Europe where application of the FCA appears to have exacerbated the gender disparity in the valuation of the cancer burden. Whereas the male/female ratio for cancer deaths was 1.31, the application of the HCA increased this ratio to 1.7, and the application of the FCA further widened the male/female burden gap to 1.9. Therefore, in countries such as Portugal (2.5), Greece (2.4) and Spain (2.2), the male/female FCA cost ratio rose above 2 even though the male/female ratio of cancer death was below 2 (Portugal: 1.8, Greece: 1.4, Spain: 1.5).

### 3.5. Premature Mortality Costs by Cancer Site

In Table 3, both the HCA and the FCA valuation approaches ranked lung (HCA: EUR 11.08 billion; FCA: EUR 0.39 billion), breast (HCA: EUR 5.04 billion; FCA: EUR 0.12 billion), colorectum (HCA: EUR 4.85 billion; FCA: EUR 0.14 billion), brain (HCA: EUR 4.2 billion; FCA: EUR 0.09) and pancreatic (HCA: EUR 3.2 billion; FCA: EUR 0.11 billion) cancer as the five most costly according to total premature mortality costs. Together, these five cancers accounted for 53% (HCA) and 54% (FCA) of the total cancer cost burden.

Cancer premature mortality costs per death according to the HCA and FCA revealed an alternative perspective on the cancer burden. According to this metric, the most costly cancers as valued by the HCA were Hodgkin Lymphoma (EUR 298,093), melanoma of the skin (EUR 252,204), brain (EUR 247,090), leukaemia (EUR 207,641) and non-Hodgkin Lymphoma (EUR 188,987). Each of these five cancers are associated with relatively high HCA/FCA cost ratios above 40, with Hodgkin Lymphoma in particular showing a cost ratio of 67.6 (the highest across all cancer sites). This is, in part, a consequence of the younger age of death associated with these cancers. The FCA included only two cancers from this list (brain (EUR 5304) and melanoma of the skin (EUR 5461)) and added to the top five most costly cancers per death head and neck (EUR 4766), kidney (EUR 5040) and oesophageal (EUR 6039) cancer.

### 3.6. Sensitivity Analysis

For the HCA (Table 4), substantial differences in the total value of lost productivity were observed following the application of minimum and maximum national economic growth rates as measured by GDP average changes. Minimum growth rates reduced costs by 22% on average compared to the base case, with a reduction of 37% in the case of Central-Eastern Europe. Costs increased by 23% on average when maximum growth rates were used with Central-Eastern Europe, again showing the highest divergence from the base case with an increase of 63%. Altering the discount rate also resulted in considerable changes, with a 0% discount rate increasing costs by 24% compared to the base case and a 6% discount rate reducing costs by 12%.

For the FCA (Table 5), substantial differences in estimated productivity costs by region and for Europe as a whole were observed following the application of country-specific minimum and maximum friction periods from the period 2008–2018. Productivity costs increased by 22% in the case of maximum friction periods for Europe. This altered the HCA/FCA cost ratio from 34 in the base case to 28. Application of the minimum friction period from 2008–2018 resulted in a decrease in productivity costs by 15% across Europe, with a resultant increase in the HCA/FCA ratio to 41. Estimated productivity costs using 2018 friction periods increased over the base case cost in line with expectations given the assumed negative relationship between vacancy duration (and hence friction period) and the unemployment rate [18], and low unemployment rates across Europe pre COVID-19 pandemic.

## 4. Discussion

The dominant valuation approach for productivity costs in the literature is the HCA; however, criticisms abound of this approach [4,22,23,24], and primary amongst these is the overestimation of productivity costs due to the assumptions that employees are not replaced following illness or death [25]. Although it has been suggested that the FCA generates more realistic estimates of productivity costs than the HCA [5], issues with regard to a standardised approach to friction period estimation across countries, and a lack of data necessary to compute accurate friction periods by country remain. It is this issue that the current study confronts by applying a standardised methodological framework for friction period estimation across Europe to estimate comparable multi-country FCA productivity cost estimates for cancer.

Our study revealed that cancer premature mortality costs for Europe were EUR 54.02 billion (HCA) and EUR 1.57 billion (FCA), indicating that HCA costs are 34 times larger than FCA costs. Estimates of the HCA/FCA costs ranged from 29.7 in Western Europe to 49.9 in Southern Europe by region, from 11 in Czechia to 65 in Ireland by country, and from 21 for prostate cancer to 67 for Hodgkin Lymphoma by cancer site. Previous reviews of FCA and HCA cancer productivity costs reveal similar variability in their cost ratios. Pike et al. [3] found that cancer studies that included mortality costs had HCA/FCA ratios between 24 and 48. By cancer site, previous single-country studies have shown HCA/FCA ratios of 49 and 73 for breast cancer in Spain [9] and Ireland [7], respectively (42 for breast cancer in our study), 56 for prostate cancer in Ireland (21 for prostate cancer in our study) [7], 55 for brain cancer in Spain (47 for brain cancer in our study) [9]) and 47 for haematologic cancer in Spain (46 in our study) [10]. Therefore, while results vary in terms of the magnitude of the HCA/FCA ratio, high-fatality diseases such as cancer do appear to generate HCA cost estimates considerably in excess of their FCA equivalents, and this result is substantiated by our estimates of cancer in Europe.

The drivers of the variability in the HCA/FCA cost ratios are varied and nuanced and arise due to a combination of underlying epidemiological features and macroeconomic features including labour market data used for valuation purposes. For example, in the case of prostate cancer where the HCA/FCA ratio is 21, the mean age of death is 59.4, which is the oldest across all cancers (average age of death for all cancers = 55.6). Consequently, there are fewer remaining productive years to cost according to the HCA diminishing the gap between the HCA and the shorter FCA costing method. Similarly, Hodgkin Lymphoma, with an HCA/FCA cost ratio of 67, had the youngest average age of death across all cancers at 46.8, allowing HCA costs to accumulate over a longer time frame. In contrast, by country, the key drivers of the low HCA/FCA cost ratio for Czechia (11), for example, were more economic in nature. Czechia had the longest friction period in the study (247 days in 2020 versus 74 days European average) and therefore had the potential to generate higher productivity costs according to the FCA. This was primarily driven by the size of the vacancy rate in Czechia (5.3% in 2020; the highest value across all EU countries), which translated into a comparatively large job vacancy stock figure. Ireland, which produced the highest HCA/FCA cost ratio of 65, had the fourth lowest friction period in Europe for 2020 (47 days), the fifth lowest age of cancer death on average (55.0 years old versus average in Europe 55.7) and, consequently, one of the highest YPPLL per number of deaths ratios in Europe (8.4, sixth highest in Europe). This range of economic and epidemiological factors indicates the complexity of factors driving productivity cost variation between both valuation methods.

Interestingly, despite the variability in HCA/FCA cost ratios highlighted across Europe, a key finding of this study was that the ranking of total premature mortality cost burden by region, country and cancer site remained generally independent of the selected valuation approach. For example, four of the top five ranked countries by total cancer productivity cost burden were the same according to both HCA and FCA, and, by cancer site, the top five ranked cancers by total cost burden were the same for both valuation approaches. This was not the case for cancer premature mortality cost per death though, where the ranking of cancer burden changed considerably by region and by cancer site depending on selected valuation approach. Traditional macroeconomic factors such as high average gross wages and low unemployment rates explain the ranking of cancer productivity cost burden by country according to the HCA (first: Switzerland, second: Denmark and third: Iceland), while the FCA highlights the speed at which an employee is replaced after leaving the workplace and ranks countries such as Belgium (first), Germany (third) and Czechia (fifth) as high cost based on the length of their friction periods (in addition to high wages and low unemployment levels in the case of Belgium and Germany).

Per death productivity costs by cancer site, on the other hand, seem to be driven by the average age of death for both the HCA and FCA with melanoma skin cancer (average age of death = 52.2) and brain cancer (average age of death = 52.2), both ranking in the top five most costly cancers according to both valuation approaches. Additionally, in the case of the FCA, the ratio of male deaths to female deaths per cancer impacts on estimated costs. Oesophageal cancer (first) and head and neck cancer (fifth) emerge as high cost per death cancers in spite of their absence from the top five most costly as measured by the HCA. Both have a male/female cancer death ratio far in excess of the average across all cancers (5.0 and 4.8, respectively, versus an average European ratio of 1.3). This represents a significant finding, and while we know that estimating economic costs can offer an interesting alternative public health perspective on the burden of diseases, we additionally highlight that the choice of valuation lens can considerably alter the reported burden of high fatality illnesses such as cancer when costs are expressed on a per death basis.

Interesting findings also emerge following an examination of the gender aspects of the differences between the HCA and the FCA. Our findings show that the FCA intensified the gender disparity in the valuation of the cancer burden. Whereas previous studies [2,11,12] have revealed the degree to which male cancer premature mortality costs exceed female costs following the application of market wages through the HCA, our results reveal how the male/female cost ratios increased on average from 1.7 for the HCA to 1.9 for the FCA. Future researchers and those implementing economic evaluations from a societal perspective should be cognisant of this gender effect and could perhaps look to use national average gross wages rather than gender specific wages to overcome this.

Following the results of previous studies, our sensitivity analysis highlighted the key role played by the length of a friction period in FCA derived productivity costs and how changes in this variable over time due to underlying macroeconomic conditions can impact productivity costs. FCA theory predicts shorter friction periods as unemployment rises [26]. For example, due to the macroeconomic effects of the COVID-19 pandemic, European unemployment rates rose considerably between 2018 and 2020, resulting in shorter friction periods in 2020 compared to 2018. The outcome of this is that the FCA productivity costs of cancer are 16% higher based on labour market conditions from the pre-COVID 2018 period compared to the COVID-19-impacted 2020 estimates. Our analyses further show that the HCA/FCA ratio increased to 41 as a consequence of using country-specific minimum friction period estimates from 2008–2018 and fell to 28 following the use of maximum estimates over this time frame. This study therefore highlights the dependence of derived FCA productivity estimates on the extant labour market conditions of a country at a point in time and the importance of updating these regularly on a national basis to obtain accurate productivity estimates over time.

Our study highlights the benefits of using a standardised methodological framework for friction period estimation, offering the potential to compare derived costs to the HCA. The findings offer valuable lessons for researchers in the range of, and discrete nature of, the drivers of costs for each approach and will aid in informing researcher and policy-maker choice between valuation approaches by providing transparency in the costing process and providing a practical example as to how costs diverge for a key constituent of indirect costs over the course of a working lifetime.

Our results also feed into the literature on cost-effectiveness analysis. Previous studies have shown that the inclusion of productivity costs in cost-effectiveness analyses have the potential to significantly impact the outcome of the analysis [27]. Recent reviews have shown that productivity cost inclusion in economic evaluations generally leads a more favourable cost-effectiveness outcome and, in a significant minority of cases, changes the incremental cost-effectiveness ratio (ICER) from positive to negative, transforming the new treatment into a cost-saving intervention [27,28]. Nevertheless, most pharmacoeconomic guidelines do not advocate a societal perspective for economic evaluations, and therefore, it is left to researcher discretion in many cases to include them. This problem is exacerbated by the lack of a universally recognized framework for calculating productivity costs; consequently, valuation approaches vary widely [4,27]. The use of a standardized methodological framework in our study to estimate FCA productivity costs based on publicly available national data sources provides a potential avenue to overcome some of the current variability noted in valuation approaches, hence strengthening the argument for their inclusion in future cost-effectiveness analyses.

Nevertheless, there are several limitations of our study. Our study is an underestimate of the productivity costs of cancer in Europe due to the explicit exclusion of other forms of cancer-related lost productivity including morbidity in the form of temporary work absences, permanent absenteeism, reduced working hours and presenteeism. The lack of a standardised database across Europe for these productivity costs for cancer resulted in their exclusion in this case. We also excluded unpaid productivity costs from our study, which have been shown to be significant for cancer [11]. This was a deliberate choice made by the authors, as non-market activity, by its nature, does not result in the need to replace employees in a firm. To aid the comparability of our estimates with previous studies of cancer productivity costs in Europe, we also excluded those individuals who die prematurely from cancer but would have worked beyond 65 years of age. Robust estimates for individuals working beyond 65 are difficult due to the limited economic data availability for wage rates, labour force participation rates and unemployment rates for this group. Our study employed gross national wage rates rather than self-reported wage rates and therefore does not reflect variations in the socio-economic background of the population under study. Nor do our estimates reflect variations in vacancy duration by occupation due to a lack of routinely collected data at a national level on vacancy stock data by sector or by occupation. Our forecasted labour market data used for the HCA was based on an average of historic trends, which may not accurately reflect future trends. This is a common shortcoming of HCA estimates, however, and one we undertook sensitivity analysis to mitigate as much as possible. Given the 2020 base case year in this study, the labour market data used for valuation purposes has been impacted the COVID-19 pandemic. We have undertaken broad ranging sensitivity analysis as a consequence by modelling the impact of future wage growth and friction period estimates on our results, and we have discussed how our FCA costs are likely influenced by these atypical macroeconomic conditions.

## 5. Conclusions

Our study provides a unique perspective on the difference between HCA and FCA estimates of cancer-related premature mortality productivity costs in Europe using a standardized methodological approach to friction period estimation. We highlight the magnitude of the difference between HCA and FCA estimates for cancer and, importantly, draw attention to the dependency of the FCA on the extant macroeconomic and labour market conditions of a country. We offer insights for researchers and policy makers into the key reasons for the differences in costs between valuation approaches while providing transparency on their application in practice.

## Figures and Tables

**Table 1:** Number of premature deaths due to cancer * (15–65 years), age of death, Years of Potential Productive Life Lost (YPPLL), cancer-related premature mortality costs (PMC) (EUR, total and per death) by Human Capital Approach (HCA) and Friction Cost Approach (FCA) and HCA/FCA ratios for Europe by region and country in 2020.

Region/Country	Deaths	Average Age at Death	YPPLL	HCA PMC (EUR Millions)	FCA PMC (EUR Millions)	HCA PMC per Death	FCA PMC per Death	HCA/FCA Ratio
***Central-Eastern Europe***	***80,970***	***55.7***	***676,240***	***5045***	***162***	***62,358***	***2001***	***31.1***
Bulgaria	6426	54.80	59,082	299	6	46,526	988	47.1
Czechia	6776	55.9	54,962	620	56	91,404	8231	11.1
Hungary	9963	56.0	80,037	656	26	65,896	2563	25.7
Poland	33,901	56.0	270,324	2152	42	63,608	1236	51.4
Romania	19,119	55.0	172,433	941	25	49,208	1291	38.1
Slovakia	4785	55.8	39,401	377	8	78,808	1620	48.6
***Northern Europe***	***51,592***	***55.5***	***442,349***	***11,165***	***280***	***216,377***	***5419***	***39.9***
Denmark	3460	56.1	27,179	1041	27	300,750	7855	38.3
Estonia	900	56.3	6945	77	2	85,475	1748	48.9
Finland	2445	56.1	19,403	536	14	219,013	5597	39.2
Iceland	133	56.2	1040	38	1	281,944	7608	37.1
Ireland	2274	55.0	20,491	617	10	271,122	4205	64.5
Latvia	1677	55.7	13,987	127	4	75,658	2182	34.7
Lithuania	2529	55.6	21,332	163	3	64,394	1138	56.6
Norway	2354	55.6	19,892	597	18	253,538	7588	33.4
Sweden	3919	55.7	32,417	1010	25	257,651	6400	40.3
United Kingdom	31,901	55.2	279,663	6960	177	218,175	5551	39.3
***Southern Europe***	***80,032***	***55.4***	***692,801***	***10,620***	***213***	***132,682***	***2660***	***49.9***
Croatia	3659	56.1	28,829	185	5	50,441	1370	36.9
Cyprus	593	54.9	5374	81	2	136,034	2875	47.3
Greece	6776	55.7	56,208	620	11	91,436	1600	57.2
Italy	32,527	55.3	283,892	5190	120	159,589	3687	43.3
Malta	192	57.3	1287	17	1	86,765	2986	29.1
Portugal	7310	54.8	67,191	812	14	111,048	1850	60.0
Slovenia	1523	56.3	11,690	156	5	102,168	3436	29.7
Spain	27,452	55.3	238,330	3560	56	129,645	2043	63.5
***Western Europe***	***124,370***	***55.8***	***1,022,535***	***27,184***	***915***	***218,624***	***7353***	***29.7***
Austria	5331	56.0	42,813	1154	43	216,417	7983	27.1
Belgium	6860	55.9	55,348	1439	71	209,742	10,387	20.2
France	42,441	55.1	376,284	7750	164	182,748	3875	47.1
Germany	55,029	56.2	430,726	12,330	507	224,067	9221	24.3
Luxemburg	279	56.3	2158	77	2	277,578	7936	35.0
Switzerland	4082	55.7	33,859	1864	38	456,884	9276	49.2
The Netherlands	10,348	56.1	81,347	2570	89	248,263	8576	29.0
***EUROPE-27+ Norway, Switzerland, Iceland, UK***	***336,964***	***55.6***	***2,833,925***	***54,015***	***1569***	***160,318***	***4656***	***34.4***

* Cancer totals include non-melanoma skin cancer.

**Table 2:** Cancer *-related premature mortality costs (PMC) (EUR, total and per cancer death) by Human Capital Approach (HCA) and Friction Cost Approach (FCA) for Europe by region and country and sex in 2020.

	Males (M)					Females (F)					Sex Ratios			
Region	HCA PMC (EUR Millions)	FCA PMC (EUR Millions)	HCA/FCA Ratio for PMC	HCA PMC per Death	FCA PMC per Death	HCA PMC (EUR Millions)	FCA PMC (EUR Millions)	HCA/FCA Ratio for PMC	HCA PMC per Death	FCA PMC per Death	M/F Deaths Ratio	M/F YPPLL Ratio	M/F HCA PMC Ratio	M/F FCA PMC Ratio
***Central-Eastern Europe***	***3208***	***107***	***29.9***	***67,387***	***2252***	***1837***	***55***	***33.6***	***55,159***	***1642***	***1.4***	***1.3***	***1.7***	***2.0***
Bulgaria	179	4	44.2	46,674	1059	120	2	52.3	46,310	883	1.5	1.2	1.5	1.8
Czechia	396	37	10.7	99,322	9326	224	19	12.0	80,113	6670	1.4	1.3	1.8	2.0
Hungary	408	17	24.7	71,973	2918	248	9	27.6	57,862	2094	1.3	1.2	1.6	1.8
Poland	1360	28	49.3	70,653	1428	792	14	55.3	54,284	982	1.3	1.2	1.7	1.9
Romania	617	17	36.9	51,683	1401	324	8	40.7	45,088	1109	1.7	1.5	1.9	2.1
Slovakia	248	5	47.0	84,601	1797	129	2	52.1	69,618	1340	1.6	1.4	1.9	2.1
***Northern Europe***	***6405***	***167***	***38.3***	***240,854***	***6297***	***4761***	***112***	***42.4***	***190,356***	***4485***	***1.1***	***0.9***	***1.3***	***1.5***
Denmark	602	16	36.9	339,945	9209	439	11	40.4	259,699	6437	1.0	0.9	1.4	1.5
Estonia	45	1	45.9	83,373	1816	32	1	54.0	88,672	1645	1.5	1.3	1.4	1.7
Finland	312	8	38.1	236,133	6208	224	5	40.8	198,926	4880	1.2	1.1	1.4	1.5
Iceland	23	1	37.1	352,591	9528	14	0.4	37.1	212,352	5716	1.0	0.9	1.6	1.6
Ireland	349	6	61.9	319,207	5162	268	4	68.3	226,620	3320	0.9	0.8	1.3	1.4
Latvia	74	2	32.5	76,248	2343	53	1	38.1	74,858	1962	1.4	1.1	1.4	1.6
Lithuania	97	2	53.5	63,219	1182	66	1	61.8	66,224	1071	1.6	1.3	1.5	1.7
Norway	341	10	32.6	282,504	8672	256	7	34.7	223,006	6445	1.1	1.0	1.3	1.4
Sweden	541	14	39.0	280,267	7176	469	11	41.8	235,683	5646	1.0	0.9	1.2	1.2
United Kingdom	4020	107	37.5	248,862	6641	2940	70	42.1	186,714	4434	1.0	0.9	1.4	1.5
***Southern Europe***	***6918***	***143***	***48.3***	***150,352***	***3110***	***3702***	***70***	***53.0***	***108,781***	***2051***	***1.4***	***1.2***	***1.9***	***2.1***
Croatia	116	3	35.6	52,578	1484	69	2	39.4	47,226	1197	1.5	1.3	1.7	1.9
Cyprus	55	1	47.2	159,771	3381	26	1	47.3	103,013	2172	1.4	1.3	2.2	2.2
Greece	418	8	54.3	105,722	1946	202	3	64.2	71,420	1115	1.4	1.1	2.1	2.4
Italy	3350	79	42.2	192,741	4564	1840	41	45.3	121,532	2680	1.1	1.0	1.8	2.0
Malta	11	0.4	28.4	98,140	3456	6	0.2	30.4	71,504	2355	1.3	1.2	1.8	2.0
Portugal	553	10	57.6	118,466	2059	259	4	66.0	97,973	1482	1.8	1.5	2.1	2.4
Slovenia	95	3	28.9	104,380	3609	60	2	31.1	98,848	3177	1.5	1.4	1.6	1.7
Spain	2320	38	60.5	141,091	2333	1240	18	70.0	112,543	1610	1.5	1.2	1.9	2.2
***Western Europe***	***17,283***	***602***	***28.7***	***243,344***	***8473***	***9901***	***313***	***31.7***	***185,690***	***5862***	***1.3***	***1.2***	***1.7***	***1.9***
Austria	747	28	26.2	250,109	9546	407	14	28.9	173,548	5994	1.3	1.1	1.8	2.0
Belgium	856	44	19.3	222,635	11,520	583	27	21.6	193,291	8941	1.3	1.1	1.5	1.6
France	5040	110	45.6	198,264	4344	2710	54	50.2	159,546	3174	1.5	1.3	1.9	2.0
Germany	7900	338	23.4	252,957	10,817	4430	170	26.1	186,157	7128	1.3	1.2	1.8	2.0
Luxemburg	50	1	34.0	297,166	8730	27	1	36.8	247,484	6715	1.5	1.4	1.8	2.0
Switzerland	1170	24	48.0	528,034	10,987	694	13	51.4	372,223	7240	1.2	1.1	1.7	1.8
The Netherlands	1520	55	27.6	294,706	10,657	1050	34	31.1	202,017	6503	1.0	0.9	1.4	1.6
***EUROPE-27+ Norway, Switzerland, Iceland, UK***	***33,814***	***1020***	***33.2***	***176,787***	***5331***	***20,200***	***549***	***36.8***	***138,686***	***3771***	***1.3***	***1.2***	***1.7***	***1.9***

* Cancer totals include non-melanoma skin cancer.

**Table 3:** Cancer-related premature mortality costs (PMC) (EUR, total and per cancer death) by Human Capital Approach (HCA) and Friction Cost Approach (FCA) for Europe by cancer site in 2020.

Cancer Site	Deaths	Average Age at Death	YPPLL	HCA PMC (EUR Millions)	FCA PMC (EUR Millions)	HCA PMC per Death	FCA PMC per Death	HCA/FCA Ratio
Head and neck	14,605	55.5	128,508	2508	72	165,007	4766	34.6
Oesophagus	10,411	56.6	76,621	1833	63	175,774	6039	29.2
Stomach	13,091	55.0	117,265	2226	60	170,057	4589	37.1
Colorectum	30,806	56.1	244,755	4850	144	157,278	4660	33.8
Liver	14,871	56.7	108,273	2249	71	151,368	4741	31.9
Gallbladder	1023	57.5	6658	105	4	102,391	3757	27.2
Pancreas	22,214	57.0	155,938	3184	109	143,128	4895	29.3
Larynx	4515	56.6	33,252	559	18	123,757	4044	30.6
Lung	83,917	57.2	569,038	11,080	389	132,088	4631	28.5
Melanoma skin	5914	52.2	70,105	1491	32	252,204	5461	46.2
Breast	29,213	53.4	310,803	5040	120	172,549	4124	41.8
Cervix Uteri	7187	51.4	90,653	1240	25	172,315	3545	48.7
Corpus Uteri	3733	57.6	23,985	307	11	82,197	2901	28.4
Ovary	9238	55.2	81,196	1180	33	127,990	3605	35.4
Prostate	5168	59.4	23,729	508	25	98,369	4776	20.6
Kidney	8318	56.3	64,481	1348	42	162,382	5040	32.2
Bladder	5730	58.0	34,632	667	25	116,449	4313	27.0
Brain and Central Nervous System	17,041	52.2	201,675	4210	90	247,090	5304	46.6
Thyroid	894	56.0	7129	140	4	156,304	4665	33.5
Hodgkin Lymphoma	885	46.8	15,338	266	4	298,093	4439	67.6
Non-Hodgkin Lymphoma	6938	54.2	68,148	1311	33	188,987	4730	39.9
Multiple Myeloma	3852	57.7	24,131	482	17	125,171	4521	27.7
Leukaemia	7777	52.4	90,158	1611	34	207,641	4398	47.1
***All cancers***	***336,964***	***55.6***	***2,833,925***	***54,000***	***1569***	***160,318***	***4656***	***34.4***
***All cancers except non-melanoma skin***	***336,070***	***55.6***	***2,826,315***	***53,900***	***1565***	***160,293***	***4656***	***34.4***

**Table 4:** Sensitivity analysis of cancer-related premature mortality costs (PMC) (EUR, total) using the Human Capital Approach with comparison to the base case *.

Region	HCA PMC (EUR Millions)	% Change from BC
**EUROPE. Discount Rate 0%**	**67,100**	**24**
Central-Eastern Europe	6350	26
Northern Europe	14,100	26
Southern Europe	13,200	25
Western Europe	33,500	23
**EUROPE. Discount Rate 6%**	**47,600**	**−12**
Central-Eastern Europe	4410	−13
Northern Europe	9770	−13
Southern Europe	9360	−12
Western Europe	24,100	−11
**EUROPE. Minimum GDP growth of each country**	**41,900**	**−22**
Central-Eastern Europe	3180	−37
Northern Europe	7850	−30
Southern Europe	8090	−24
Western Europe	22,800	−16
**EUROPE. Maximum GDP growth of each country**	**66,600**	**23**
Central-Eastern Europe	8250	63
Northern Europe	16,400	46
Southern Europe	12,100	14
Western Europe	29,900	10

* Base case: up to 65 years old, Discount Rate = 3.5%, average Gross Domestic Product (GDP) growth of each country.

**Table 5:** Sensitivity analysis of cancer-related premature mortality costs (PMC) (EUR, total) using the Friction Cost Approach with comparison to the base case *.

Region/Country	Friction Periods in Days	FCA PMC (EUR Millions)	% Change from the Base Case	HCA/FCA Ratio
**EUROPE. Friction period = 2018**	**79**	**1823**	**16**	**30**
Central-Eastern Europe	105.2	173	7	29
Northern Europe	69.2	342	22	33
Southern Europe	61.1	225	6	47
Western Europe	91.7	1083	18	25
**EUROPE. Minimum friction periods (2008–2018)**	**55.8**	**1331**	**−15**	**41**
Central-Eastern Europe	60.8	100	−38	50
Northern Europe	54.6	265	−5	42
Southern Europe	44.9	204	−4	52
Western Europe	66.7	762	−17	36
**EUROPE. Maximum friction periods (2008–2018)**	**85.9**	**1920**	**22**	**28**
Central-Eastern Europe	106.4	174	8	29
Northern Europe	74.2	346	24	32
Southern Europe	73.1	253	19	42
Western Europe	92.5	1146	25	24

* Base case: Friction periods in 2020 for each country.

## Data Availability

Publicly available datasets were analysed in this study. These data can be found here: https://gco.iarc.fr/today/data-sources-methods (accessed on 7 January 2022) and https://ec.europa.eu/eurostat/data/database (accessed on 8 January 2022).
